# Evaluation of Changes Associated with an Educational Intervention on Basic Life Support and Airway Obstruction Among Schoolchildren Aged from 11 to 18 Years Old in the Island of La Palma (Canary Islands)

**DOI:** 10.3390/nursrep16040138

**Published:** 2026-04-15

**Authors:** Sofía Martínez-León, Alba Francisco-Sánchez, Beatriz Rescalvo-Arjona, Pedro Ruymán Brito-Brito, Martín Rodríguez-Álvaro

**Affiliations:** 1 Primary Health Care, La Palma Health Services, The Canary Islands Health Service, 38713 Breña Alta, Spain; smarleo@gobiernodecanarias.org (S.M.-L.); bresarj@gobiernodecanarias.org (B.R.-A.); mrodalve@gobiernodecanarias.org (M.R.-Á.); 2 Nursing Department, Nursing Faculty, University of La Laguna, 38200 Santa Cruz de Tenerife, Spain

**Keywords:** cardiopulmonary resuscitation, basic life support, cardiovascular diseases, school health training

## Abstract

Cardiopulmonary arrests are time-dependent emergencies where survival rates are quickly reduced without early intervention. BLS training programmes for teachers and schoolchildren must be mandatory, as they not only improve efficacy when performing the manoeuvres but also enhance willingness to do so. **Background/Objectives**: To analyse changes in knowledge and whether they are sustained in time after a theoretical–practical intervention led by nurses. To objectively analyse the quality of chest compressions according to the students’ group and age. **Methods**: A quasi-experimental study without a Control Group and with three measuring instances: Baseline (T0), Immediate post-intervention (T1) and at three months (T2). Knowledge was assessed by means of an ad hoc questionnaire; in turn, the chest compressions were evaluated using a mannequin with feedback. The longitudinal changes were analysed based on paired discordant answers. Descriptive analyses according to age and schooling level were performed. **Results**: There were 982, 756 and 509 students at T0, T1 and T2, respectively. A total of 206 records were paired at all three measuring moments. The intervention was associated with an increase in knowledge test scores, which is preserved at three months. Most of the questionnaire items presented positive changes or remained unchanged. The significant difference was maintained in 8 of the 10 questions. As for the compressions, a significant and positive correlation was found between age and overall score, depth and rate. The comparative analysis between the Lower Secondary Education and Higher Secondary Education groups found significant differences in those same variables, as well as a difference in release. **Conclusions**: Altogether, the results of this study contribute evidence about the effectiveness of BLS training among adolescents in a real-world context, underscoring the need for ongoing and age-adapted interventions.

## 1. Introduction

Cardiopulmonary arrest (CA) is a time-dependent emergency in which survival rapidly decreases without early intervention. Most cases take place in out-of-hospital settings and only a minority of the victims are offered Cardiopulmonary Resuscitation (CPR) by bystanders. Foreign-Body Airway Obstruction (FBAO) is another preventable cause of death, especially in the young population. The evidence shows training deficits in terms of Basic Life Support (BLS) and FBAO and recommends implementing systematic training programmes in educational centres [1]. There are doubts as to the best age for long-term learning and knowledge retention, which justifies assessing educational interventions with a time-sensitive follow-up.

CAs are produced by the interruption of the cardiac mechanical activity and can be diagnosed in the absence of consciousness, pulse and breathing [1]. Around 60–80% of all CAs take place in out-of-hospital settings, where only one out of ten patients manage to survive [2]. Neurological sequelae are produced ten minutes after a CA, and mortality is practically 100% twelve minutes after its beginning [3].

CPR is understood as the set of manoeuvres targeted at reverting a CA situation by substituting the victim’s pulmonary and cardiac functions [1,3]. Currently, CPR manoeuvres are put into practice in one out of five out-of-hospital CAs [2].

On the other hand, FBAO is usually urgent and a potential cause of death, especially in the youngest and oldest individuals [4,5,6]. The result will depend on how fast the airway obstruction relief manoeuvres are implemented. As for the victims’ prognoses, one of the most determinant factors is the role that lay people may play. Bystanders’ fast and effective actions can be the key to survival [4].

In general, the response time for medical responders to reach the scene of the emergency is usually longer than the time a lay person might provide any help. Fast and effective responses by unspecialized but trained personnel might improve the survival rate to 50% [2,3].

When assessing what schoolchildren [7], teachers and parents [3] know about this topic, inadequate training is mainly evidenced in a lack of understanding about CPR manoeuvres and how to use semi-automatic defibrillators. This lack of knowledge means the population is not confident enough to offer help.

In the Canary Islands, Royal Decree No. 89/2014 dated 1 August establishes that First Aid training is proposed in the Primary Education basic curriculum [8]. However, teachers’ training in this scope is not mandatory in Spain. Currently, only 30% of Primary and/or Childhood Education university degrees include First Aid in their curricula [9].

BLS training programmes for teachers and schoolchildren must be mandatory, as they not only improve efficacy when performing the manoeuvres but also enhance the willingness to do so [8]. In addition, they state that the schoolchildren that are provided with this training pass on what they learn to the people closest to them [10].

As for the age at which this type of training should be offered, there is still no consensus in the literature. According to the American Heart Association [AHA], schoolchildren aged 4 years old should be taught about the emergency telephone number [10]. From the age of 5–7 years old, they should be instructed on how to call the emergency services, on BLS skills, on the recovery position and on how to operate Semi-Automatic External Defibrillators (AEDs). However, the “Kids Save Life” study indicates that this training can be initiated at age 9 [8,10].

As for chest compressions, the age threshold is not clear since, while the AHA recommends that it might be started at 11–16 years old, the International Liaison Committee on Resuscitation (ILCOR) tells us that it should be offered to individuals over the age of 10 [11]. In turn, there is also no consensus about the age at which FBAO training should be initiated: the literature indicates that children between 11 and 12 years old should be aware of the differences between mild and severe FBAOs and that they should learn to perform abdominal compressions from the age of 13–14. Likewise, some authors suggest that first aiders’ anthropometric characteristics are more relevant than their age for them to properly perform the manoeuvres [4].

It is recommended that training in educational centres should be at least 2 instructional hours throughout the school year, and that its content should include the PAH (“Protect, Alert and Help”) protocol and the “Survival Chain”: early recognition, asking for help, CPR by bystanders, early defibrillation and the arrival of advanced life support services [2,3,11]. It is also important to sustain training over time, carrying out brief refresher sessions lasting 5–15 min by means of pills (short modules) or videos, among other techniques [2,3].

It is worth noting that discrepancies are also found as for who should lead the training. This has generally been up to health professionals. However, articles such as the Granada Cervantes Model [3] and Kids Save Life [10] state that training led by professors previously coached by health personnel yields better results in activating emergency services, in opening the airway and in the compression/ventilation sequence [3,8,10].

It is for this reason that it is necessary to conduct studies about Basic Life Support education in school settings, as well as to assess its impact and to objectively know the quality of the resuscitation manoeuvres in order to respond to the discrepancies evidenced in the scientific literature.

The main objective of this research is to assess the efficacy of an educational intervention on BLS and FBAO among schoolchildren attending Lower Secondary Education (LSE) and Higher Secondary Education (HSE) on the island of La Palma (Canary Islands). The secondary objectives set forth are as follows: to improve the students’ knowledge and skills; to measure the quality of the chest compressions; to assess the acquisition of competencies like PAH, recognising breathing and consciousness states, activating the 112 emergency services number and how to act when facing an FBAO situation; and to analyse learning retention at three months.

The study’s working hypothesis is that a brief intervention led by nursing personnel in school settings can significantly improve the students’ theoretical knowledge, which will be maintained over time.

## 2. Materials and Methods

### 2.1. Design and Sampling Method

A quasi-experimental study without a Control Group and with three measuring instances was designed: Baseline (T0), Immediate post-intervention (T1) and at three months (T2).

As a quasi-experimental study without a Control Group, this design allows the identification of associations but does not permit causal inference.

### 2.2. Population, Sample and Sample Selection

According to the information provided by the Education Council of the Canary Islands Government, in November 2023 the number of students enrolled in public educational centres at the beginning of the school year on the island of La Palma was 2593 and 1107 LSE and HSE students, respectively.

Initially, a theoretical sample calculation stratified by educational level was made, estimating a sample comprising 335 and 286 LSE and HSE schoolchildren, respectively (95% confidence level and 5% error margin).

The final sample was selected by means of non-probability sampling.

Inclusion criteria comprised students aged 11–18 years enrolled in participating schools. Exclusion criteria included absence during assessment sessions or inability to complete the questionnaire.

### 2.3. Flow of Participants

The students’ theoretical knowledge was assessed at three timepoints: Pre-intervention (T0), Immediate post-intervention (T1) and at three months (T2). The data were collected by means of self-coded questionnaires, which required pairing the records for the longitudinal analysis of the changes.

The objective assessment of the chest compressions was made during the practical component of the intervention.

### 2.4. Nursing Intervention Design and Preparation

The educational intervention was developed for 55 min, including theoretical contents and supervised practices on airways, breathing, recovery position (RP), FBAO manoeuvres and chest compressions with a mannequin.

Both the intervention and the questionnaire were designed by a multidisciplinary team comprising three professionals: a Generalist nurse, a nurse and a physician, all specialised in Family and Community Care. All three team members are instructors of the National Plan for Cardiopulmonary Resuscitation from the Spanish Society of Critical Intensive Medicine and Coronary Units.

The questionnaire was not formally validated. Instead, content validity was ensured through expert review by a multidisciplinary team of instructors, and a pilot test was conducted to assess readability and comprehension for the target age group. Questions were single-best-answer (4 options per item). Scoring consisted of 1 point per correct answer with no pre-established cut-off point. No negative marking or weighting was applied for incorrect responses.

The intervention was based on the recommendations in force at the time the study was conducted [12].

It was implemented at a minimum and maximum instructor/participant ratio of 1:5 and 1:10, respectively. The intervention has a theoretical component and a practical one, distributed as follows:

1. Workshop theoretical component. Time: 25 min.
2. Practical component. Time: 30 min.

The schoolchildren were divided into two groups, with a maximum instructor/participant ratio of 1:10.

2.1. The following was practised in this phase of the intervention: airway obstruction relief; seeing, hearing and feeling; as well as the recovery position.
2.2. The FBAO algorithm.
2.3. Chest compressions with simulation mannequins offering no feedback.
2.4. In a reduced group, the efficacy of the chest compressions was objectively evaluated. For operational and resource availability reasons, this practical assessment was only implemented with a subgroup of students. Participants were allocated to the practical assessment subgroup based on their seating position within the classroom. When the feedback mannequin, an extra monitor for data collection, and suitable room conditions were available, students were assigned to one of three practice stations according to their natural seating order. No formal randomization was applied; allocation was based on classroom logistics and resource availability. All CPR quality measurements were recorded over a standardised 60 s interval.

Intervention pilot test: Before initiating the project, the intervention and questionnaire were pilot tested with several groups of people of similar ages to adjust the times and dynamics. It was not necessary to adapt the protocol, or the schedule intended, in any way while developing the project. To ensure reliability of the intervention, at least two research team members were always present while it was being developed.

Including a re-evaluation of the participants, the intervention was implemented between September 2024 and June 2025.

### 2.5. Variables

Data collection and analysis were done separately. To assess the theoretical knowledge, the instrument used to collect and measure the variables included in the study was a self-applied questionnaire. For the practical component, the feedback from a Little Anne CQPR Laerdal Medical mannequin (c) was recorded.

Main variables:

Related to the theoretical component:

Basic CPR and First Aid knowledge: questionnaire consisting of 10 items with four answer options each.

Related to the practical component: the QCPR App (along with a Little Anne CQPR mannequin) was used to collect these variables.

Quality of the chest compressions:

Overall score: percentage of the overall quality of the CPR manoeuvres performed (the calculation considers the release, depth, frequency, and rate).

Release: Percentage of compressions in which the chest was allowed to completely return to its normal position (full release/chest recoil).

Depth: Percentage of compressions that reached the proper depth (at least 5–6 cm in adults).

Frequency: Frequency in a minute.

Rate: Percentage of compressions within the target frequency range.

Secondary variables:

Gender: Female/male/other/fluid.

Age.

Centre.

Year/group.

Previous training in CPR.

The study was anchored in the Transparent Reporting of Evaluations with Non-randomised Designs (TREND) checklist for non-randomised trials [13].

### 2.6. Data Collection Procedure

The study was conducted in compliance with current European and Spanish laws and regulations regarding personal data processing, communication and transfer, adhering to the provisions set forth in Regulation (EU) 2016/679 of the European Parliament and Council, dated 27 April 2016 (GDPR), and in Spanish Organic Law 3/2018 on Personal Data Protection and Guarantee of Digital Rights, dated 5 December. The study was approved by the La Palma Health Services Management Office and by the provincial Ethics Committee on 24 May 2024 (CHUC_2024_34).

The students were anonymised with an alphanumeric code, thus ensuring data confidentiality and safety as per the current regulations. The database was hosted on one of the institution’s servers, which can only be accessed by the lead researcher and her collaborators. The contents included in such database will remain in this device for the next 5 years, with a security mechanism that will request a username and password to access the data.

Given the non-invasive nature of the intervention and its alignment with the official curriculum (Royal Decree 89/2014), the requirement for written informed consent was waived. Families were informed, participation was voluntary (with no penalty for non-participation), and verbal assent was obtained from all students. All procedures complied with national regulations on educational research involving minors and data protection standards.

### 2.7. Data Analysis

Three analytical approaches were applied: (1) cross-sectional analyses including all available participants at each time point; (2) partially paired analyses using linked pre–post data (T0–T1, T0–T2, T1–T2); and (3) fully paired longitudinal analyses restricted to students with complete traceable data across all three time points (T0–T1–T2), allowing the evaluation of intra-individual changes and learning retention.

The sample was described with frequency of categories for the nominal and ordinal variables and with mean standard deviation or median percentiles for the scalar ones, according to their distribution. The following techniques were used to study correlations: Pearson’s Chi-squared test, Mann–Whitney’s U test and Spearman’s rank correlation coefficient, according to the type of variable, relationship and distribution normality.

As best suited, the Friedman test, Wilcoxon signed-rank test or McNemar test were used to compare and analyse the pre-/post-intervention differences based on the variables stated in the objectives.

In order to assess the strength and direction of the relationships between variables and the size of the differences, r [r = z/√N] was used for the bivariate comparisons and Kendall’s test for concordance in repeated measurements. The effect size was assessed following the reference thresholds proposed by Cohen (small: 0.2; moderate: 0.5; large: 0.8) [14].

A post hoc power analysis was performed using G*Power (v.3.1.9.7) [15]. For a Wilcoxon signed-rank test with a final sample of n = 206 (alpha = 0.05, effect size d = 0.3), the achieved power was >0.95, confirming the study remains sufficiently powered to detect significant differences despite the attrition rate.

All the bilateral tests were performed at significance levels of alpha < 0.05, with the aid of SPSS 29.0.

## 3. Results

### 3.1. Sample Profile

The initial sample (T0) comprised 982 students (723 attending Lower Secondary Education, 259 attending Higher Secondary Education). A total of 756 students took part in the Immediate post-intervention assessment; in turn, 509 valid answers were obtained in the evaluation at three months.

The reduction in traceable cases was primarily due to inconsistencies in self-generated identification codes rather than actual dropout. This precluded proper longitudinal pairing of all records across all measurement points. Therefore, the sample loss is considered an administrative flaw and not as the participants withdrawing from the educational intervention, given that all students received the intervention and more than 500 completed the third measurement.

**Figure 1:**
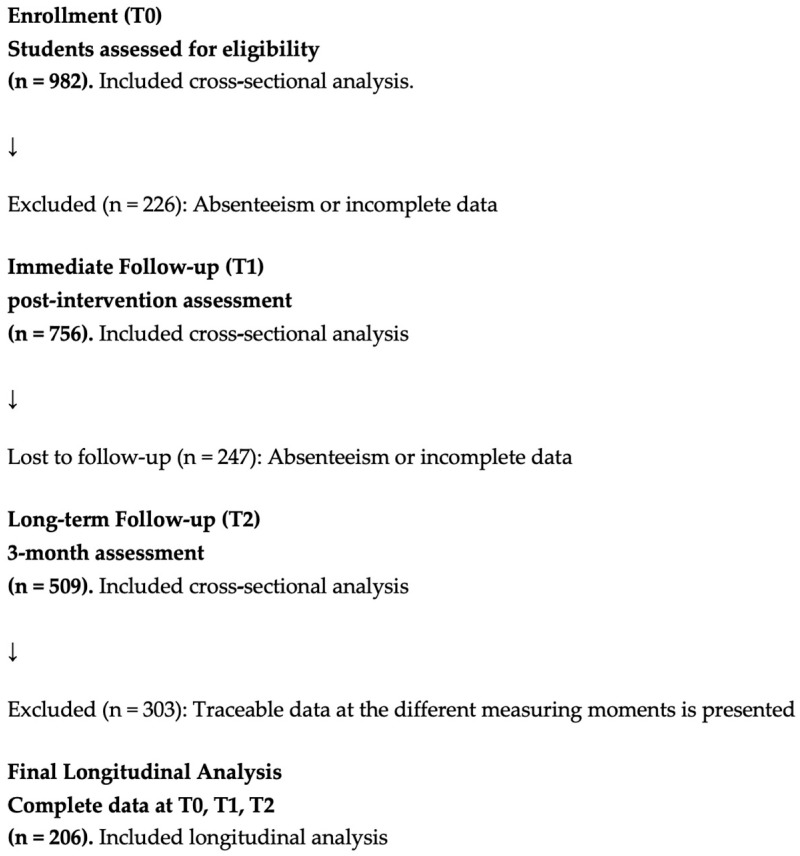
Participant flow diagram across study phases (T0–T2).

The total number of students with full and traceable data at the different measuring points is presented in Table 1 and Figure 1.

### 3.2. Cross-Sectional Analysis

To evaluate if the traceability loss introduced any selection bias, the Pre-intervention (T0) scores obtained by the group with full and traceable data at all three instances (n = 206) were compared to the rest, being higher at the Pre-intervention timepoint (*p* = 0.002).

For descriptive purposes, a sensitivity analysis was performed in G*Power [14] with the final T0-T1-T2 sample size (α = 0.05; power = 0.80). The detectable size effect was estimated under different inter-measure correlation scenarios (*p* = 0.2–0.5). In all of them, the final sample size obtained for the analysis of changes (n = 206) allowed for the detection of small size effects (f = 0.09–0.11).

The intervention was applied to a total of 982 students attending educational centres on the island of La Palma. They were 723 LSE students and 259 HSE students aged between 11 and 18 years old.

Table 2 shows the theoretical scores obtained by the students at the three timepoints, divided by type of teaching and reflecting the mean age in each year.

The Boys–Girls, LSE–HSE students and previous training scores were compared at the three measuring timepoints using the Mann–Whitney test, as Shapiro–Wilk’s test showed a significant deviation from normality (*p* < 0.001):

- Boys–Girls. The girls scored slightly higher at the three measuring timepoints. There were no significant differences at the first measuring instance (*p* = 0.84) or in the one made at three months (*p* = 0.38). However, there was a significant difference at the immediate post-test measuring timepoint (*p* = 0.033).
- LSE–HSE. The HSE students scored significantly higher at the three measuring timepoints (*p* < 0.001).
- Previous training. The students with previous training reached higher mean scores at T0 (*p* < 0.001) and at T1 (*p* < 0.001).

### 3.3. Comparison in a Partially Paired Sample (T0–T1, T0–T2, T1–T2)

T0–T1 (n = 628). There was a significant increase in the score immediately after the intervention (Z = −18.69; *p* < 0.001), with a large size effect (r = −0.75).

T0–T2 (n = 279). Likewise, there was a significant increase in the score at three months when compared to the Baseline measuring timepoint (Z = −10.24; *p* < 0.001), with a large size effect (r = −0.61).

T1–T2 (n = 233). A significant reduction was identified in the score at three months when compared to the value obtained immediately after the intervention (Z = −2.08; *p* = 0.04), with a small size effect (r = −0.12).

### 3.4. Analysis of the Answers Given to the Questionnaire

The analysis performed with the McNemar test about the evolution of the answers given to each question, comparing timepoints T0–T1 and T0–T2, is shown in Table S1. Significant changes were noticed in each of the questions between timepoints T0 and T1, with more students giving correct answers.

In order to ease their interpretation, the percentage of correct answers at each measuring timepoint and the longitudinal changes between the T0–T1 and T0–T2 timepoints are presented, calculated based on the discordant answers (Table 3). When comparing T0 to T2, most of the questionnaire items (9 out of 10) presented positive changes or remained unchanged. The significant difference was maintained in 8 of the 10 questions. At three months, the question relating to the RP presented a negative net change.

The percentage of correct answers in each of the questions at the three study timepoints when comparing LSE and HSE students is presented in Table S2. Regardless of the measuring timepoint, the rate of correct answers was higher for all the questions among the HSE students. It is noteworthy that five questions failed to reach 70% correct answers in the Immediate post-intervention period and that the highest percentage of correct answers to the question related to the effectiveness of the chest compressions was detected in the measurement made at 3 months.

As for the comparison between the answers given at timepoints T1 and T2, two questions reached significantly lower scores in the measurement made at three months, observing more correct–incorrect answer changes: the question related to when you should stop if you have initiated the CPR manoeuvres, and the one related to the manoeuvre for a person that is choking (Table S3). The net difference in discordant answers is shown in Table S4.

### 3.5. Longitudinal Analysis of Intra-Individual Changes (T0-T1-T2)

To assess intra-individual changes and the retention of what was learned, a longitudinal analysis of the sample with full and correct data at all three timepoints was performed. The number of students that answered all three questionnaires ensuring traceability was 206, most of them (n = 131) attending LSE.

This subgroup obtained higher values than the rest in the assessments, with a significant difference in the Pre-intervention measuring instance but not in the other two (T0_206: Median: 6; IQR: 3; *p* = 0.002; T1_206: Median: 8; IQR: 2; *p* = 0.257; T2_206: Median: 8; IQR: 3; *p* = 0.271).

Friedman’s test was performed to compare the scores obtained by these students at T0-T1-T2. The result was significant (*p* < 0.001), with a small–moderate size effect (Kendall’s W = 0.27). The effect size was larger among the HSE students (n = 75; Kendall’s W = 0.32) than in their LSE counterparts (n = 131; Kendall’s W = 0.25).

The comparisons by pairs using the Wilcoxon signed-rank test showed significant differences (*p* < 0.001) between timepoint T0–T1 (z = −9) and T0–T2 (z = −8.7) but not between T1 and T2 (*p* = 0.095, z = −1.7). This indicates that, although the effect size was small–moderate, it was maintained at three months.

### 3.6. CPR Quality Analysis (n = 116)

The quality of the resuscitation manoeuvres was objectively measured in 116 students. The total scores obtained, and their categorization, are shown in Table 4. Almost 9 out of every 10 HSE students reached optimum scores, whereas this is true in almost 7 out of every 10 LSE students.

A significant and positive correlation was found between age and overall score (Spearman: 0.385; n = 114; *p* < 0.001), depth (Spearman: 0.366; n = 114; *p* < 0.001) and rate (Spearman: 0.445; n = 109; *p* < 0.001). The comparative analysis between the Lower Secondary Education and Higher Secondary Education groups found significant differences in those same variables, as well as a marginal difference in release (*p* = 0.05). However, no significant differences were detected in frequency (Table 5).

The score obtained at the Immediate post-intervention timepoint was compared to the CPR quality total score. A positive and weak correlation was observed between the score and CPR global quality (Spearman’s r = 0.31; *p* = 0.002).

When the descriptive analysis by academic year was performed, it was evidenced that the worst score was obtained in the LSE first year, especially in the rate or percentage of compressions, which remains within the target frequency range. It draws the attention that, despite reaching a higher global score, HSE first- and second-year students obtain the worst score in the release item (Table S5).

Although the percentage of boys with an optimum global score was higher (82%) than among the girls (70%), no significant differences were found by gender in the study variables: overall score, release, depth, frequency and rate.

## 4. Discussion

The results of this study show that a brief intervention on BLS and FBAO is associated with a significant improvement in theoretical knowledge, with partial retention at three months. They also suggest that, in our context and already by the age of 12, most of the students may be able to maintain adequate resuscitation quality for one minute.

The findings coincide with the previous literature: training in CPR and First Aid in school settings effectively promotes changes in knowledge and skills among adolescents. In addition, this type of intervention is usually simple and cost-effective, which is why its global implementation is promoted [15].

The current evidence supports the idea that initiating CPR training at an early age increases the rate of CPR manoeuvres performed by bystanders and, consequently, that it improves survival [16]. Mass BLS education is positively associated with survival at 30 days after a cardiopulmonary arrest, as evidenced by a cohort study that included more than 50,000 incidents [17].

There is no clear consensus regarding the age at which adolescents can perform optimum resuscitation manoeuvres; however, the usual recommendation for CPR training is from the age of 12. In our study, the quality of the compressions improved significantly with age, in line with previous surveys.

The correlation between age, weight, height, BMI and resuscitation quality has been observed. Suss-Havemann reported that 12-year-old students presented approximately 20% lower rates than their 14-year-old counterparts in depth, compression frequency and pauses [18]. Other studies signal 12 years old as the age at which schoolchildren can perform chest compressions [19]; however, 13 years old seems to be the minimum age to reach optimum CPR quality [20].

According to the findings of our study, almost half of the students can maintain optimum CPR manoeuvres at age 12, rising to 8 out of every 10 at 13 years old. From that age on and with a mean weight of 50 kg, people have sufficient physical capacity to apply CPR techniques and present sufficient cognitive development levels to assimilate the importance of cardiopulmonary arrests [21].

Despite obtaining a better global score, older students performed worse in chest release. A study published in 2020 that included 567 students aged from 8 to 12 years old evidenced that the continuity and depth of the compressions improved with age, whereas the percentage of compressions with proper release was significantly reduced, which coincides with the results obtained in the current study [22]. This suggests that more physical strength might induce sustained over-compression, reducing full chest recoil, an important aspect for coronary perfusion. The need to not only emphasise depth but also chest recoil is evidenced, especially among older students.

In our study, the measurements were made in one minute. In resuscitation, schoolchildren’s performance was significantly reduced after one minute—a reason why it might be reasonable to replace the first aider, although the potential added risk is still to be explored [23].

In relation to the theoretical content, the possibility of devoting less time to concepts that have proved to present a ceiling effect should be evaluated in the future, such as the emergency services number. The high percentage of correct answers reduced the number of discordant pairs. Knowledge about FBAO and when to stop CPR was significantly reduced at three months. Using information pills or brief lessons can mitigate this. Utilising computer programs or mobile apps improves knowledge levels [24].

On the other hand, the fact that the highest percentage of correct answers to the question related to the effectiveness of the chest compressions was detected in the measurement at 3 months could potentially be explained by the teachers’ corrections during the practical training component. However, as this was not directly measured, this remains a hypothesis that requires further investigation. In addition, the weak correlation observed between theoretical knowledge and practical performance underscores the importance of practical training programmes in this scope.

This study is not exempt from limitations. The absence of validated tools to measure knowledge about BLS and FBAO that combine the quality of the resuscitation manoeuvres hinders comparisons with other studies. The absence of full psychometric validation limits comparability and should be considered when interpreting findings. Furthermore, the loss to follow-up was not random and may have led to an overestimation, although cross-sectional analyses remain robust due to the large sample size. In addition, the absence of a Control Group and the non-probability sampling method can impair the external validity of the results. Future studies should incorporate controlled designs and use validated tools that allow us to confirm these findings in other contexts. A remarkable limitation was the high rate of losses between measuring instances, due to errors in the identification codes self-generated by the students. Notably, this subgroup exhibited significantly higher Baseline scores compared to the rest of the sample, suggesting a potential selection bias. This limitation may lead to an overestimation of long-term knowledge retention, as the longitudinal analysis relies on a more proficient subset of participants. Nevertheless, the unlinked questionnaires showed similar trends in the cross-sectional analyses—the reason why we interpret the loss to be random and not reflective of any withdrawal or disinterest bias. Despite this loss, the longitudinal analysis and the effect size calculations contribute robustness to the analysis of the intra-individual changes.

These findings should be interpreted cautiously given the quasi-experimental design, potential selection bias, and measurement limitations.

## 5. Conclusions

The findings should be interpreted cautiously given the quasi-experimental design, absence of a Control Group, significant attrition, and potential selection bias. However, altogether, the results of this study contribute evidence about the effectiveness of BLS training among adolescents in a real-world context, underscoring the need for ongoing and age-adapted interventions. Systematically incorporating these programmes with nurses leading them might contribute to improving community responses to vital emergencies.

## Figures and Tables

**Table 1:** Students with full and traceable data at the different measuring points.

	T0	T1	T2	T0–T1	T0–T2	T1–T2	T0–T1–T2
Valid	982	756	509	628	279	233	206

**Table 2:** Scores corresponding to the pre-/post-intervention and 3 months after the intervention.

Year		T0	T1	T2
(Mean Age)				
1st LSE (11.7)	n	128	88	46
	Mean	3.8	6.6	5.7
	Median	4	7	6
	Standard Deviation	1.9	1.9	2.2
	IQR	3	3	3
2nd LSE (12.7)	n	117	129	78
	Mean	4.5	6.7	6
	Median	5	7	6
	Standard Deviation	1.7	2.2	2
	IQR	3	2	2
3rd LSE (14.6)	n	261	172	133
	Mean	5.5	7.5	7.3
	Median	5	8	8
	Standard Deviation	2.2	2	1.8
	IQR	3	3	3
4th LSE (15.4)	n	217	148	69
	Mean	6	7.6	7.8
	Median	6	8	8
	Standard Deviation	1.9	1.9	1.9
	IQR	2	2	2
1st HSE (16)	n	173	145	162
	Mean	6.2	8.5	8
	Median	6	9	8
	Standard Deviation	1.7	1.6	1.7
	IQR	2	2	2
2nd HSE (17)	n	86	74	21
	Mean	6.1	8.4	7.9
	Median	6	9	8
	Standard Deviation	1.9	1.4	1.4
	IQR	2	1	2
Total HSE (16.6)	Valid	259	219	183
	Mean	6.4	8.4	8.3
	Median	6	9	9
	Standard Deviation	1.7	1.6	1.7
Total LSE (14)	IQR	1	2	2
	Valid	723	537	326
	Mean	5.2	7.3	7
	Median	5	7	7
	Standard Deviation	2.2	2	1.8
	IQR	3	3	2
Total	Valid	982	756	509
	Mean	5.8	7.7	7.4
	Median	6	8	8
	Standard Deviation	2	2	1.9
	IQR	3	2	3

**Table 3:** Percentage of correct answers and net difference in discordant answers (T0–T1, T0–T2).

	T0 (n = 982)	T1 (n = 756)	T2 (n = 509)	T0–T1 (n = 628)		T0–T2 (n = 279)	
Questions	(%)	(%)	(%)	Net Change (%)	*p* *	Net Change (%)	*p* *
What does the PAH acronym mean?	45.7	67.6	66.9	24.5	<0.001	24.1	<0.001
What is the national emergency services number in Spain?	89.6	96.4	95.5	7.8	<0.001	3.9	0.350
In the recovery position (RP)…	51.9	65.0	52.9	16.6	<0.001	−2.1	0.659
What is the proper position for the first aider’s hands in cardiac massage?	70.5	88.9	86.3	20.6	<0.001	10.4	<0.001
How would you verify whether the victim is breathing or not?	88.0	94.3	92.4	6.8	<0.001	5.0	0.045
If you come across an unconscious person, which of the following measures should you implement first?	39.2	71.6	66.4	35.9	<0.001	22.1	<0.001
Chest compressions are effective if:	49.8	64.1	70.7	20.9	<0.001	19.6	<0.001
The first manoeuvre for a person that is coughing and choking is:	22.7	61.6	54.7	39.6	<0.001	35.0	<0.001
An unconscious person that is breathing needs:	58.9	76.2	82.0	17.1	<0.001	14.3	<0.001
If you have initiated CPR, when should you stop?	26.9	62.0	58.2	39.3	<0.001	28.9	<0.001

The T0, T1 and T2 values correspond to independent cross-sectional values. T0–T1, T0–T2: the net change was calculated exclusively based on the paired discordant answers at the different moments. The results are presented as the net difference in discordant answers. * *p*-values < 0.05 were considered statistically significant.

**Table 4:** CPR quality. Categorization by scores.

Overall Score	Total (116)	HSE (n = 46)	LSE (n = 70)
Optimum (≥90)	75	87	67.1
Acceptable (75–89)	12.9	6.5	17.1
Needs to be improved (<75)	12.1	6.5	15.7

**Table 5:** CPR quality. Mean scores by item and comparison between stages: LSE/HSE.

	Total		LSE (n = 70)		HSE (n = 46)		Mann-Whitney’s U	Z	r	Sig *
	Median	IQR	Median	IQR	Median	IQR				
Score	96	11	94	16	98	5	949.5	−3.8	−0.35	<0.001
Release	96	17	99	18	89.5	16	1272.0	−1.9	−0.18	0.050
Depth	99	11	96	23	100	2	900.0	−4	−0.37	<0.001
Frequency	116	13	118	14	114.5	11	1127.5	−1.5	−0.15	0.129
Rate	65	56	48	58	83	45	891.0	−3.6	−0.34	<0.001

Measurements recorded over a 60 s interval. Statistical significance determined by Mann–Whitney U test (Z = LSE-HSE). IQR: Interquartile Range; r: effect size (Z/√N). * *p* < 0.05 considered statistically significant.

## Data Availability

The original contributions presented in this study are included in the article/Supplementary Materials. Further inquiries can be directed to the corresponding authors.

## Supplementary Materials

The following supporting information can be downloaded at: https://www.mdpi.com/article/10.3390/nursrep16040138/s1, Table S1: Comparison between the previous correct answers and the ones given at the Immediate post-intervention and at three months; Table S2: Percentage of correct answers given to each of the questionnaire questions, by teaching level (LSE-HSE); Table S3: Comparison between correct answers at the Immediate post-intervention and at three months; Table S4: Net difference in discordant answers (T1–T2); Table S5: CPR quality. Mean score by item and comparison between academic years.

## Public Involvement Statement

No public involvement in any aspect of this research.

## Guidelines and Standards Statement

This manuscript was drafted against the TREND (Transparent Reporting of Evaluations with Non-randomised Designs) reporting guidelines for non-randomised evaluations of behavioural and public health interventions.

## Abbreviations

The following abbreviations are used in this manuscript:

AED	Automated External Defibrillator
BLS	Basic Life Support
CA	Cardiopulmonary Arrest
CPR	Cardiopulmonary Resuscitation
FBAO	Foreign-Body Airway Obstruction
HSE	Higher Secondary Education
IQR	Interquartile Range
LSE	Lower Secondary Education
PAH	“Protect, Alert and Help” protocol
RP	Recovery Position
T0	Baseline/Pre-intervention measurement
T1	Immediate post-intervention measurement
T2	Three-month follow-up measurement
TREND	Transparent Reporting of Evaluations with Non-randomised Designs
