# Supplementary material for: Evaluation of Changes Associated with an Educational Intervention on Basic Life Support and Airway Obstruction Among Schoolchildren Aged from 11 to 18 Years Old in the Island of La Palma (Canary Islands)
Source: Nurs Rep. 2026 Apr 15;16(4):138. doi: 10.3390/nursrep16040138 (PMC13118523; doi:10.3390/nursrep16040138)
Supplement: Supplementary file 1 [file nursrep-16-00138-s001.zip › nursrep-4221505-supplementary.pdf]

Assessment of an educational intervention on Basic Life Support and Airway Obstruction among schoolchildren aged from 11 to 18 years old in the island of La Palma (Canary Islands)

SUPPLEMENTARY MATERIAL

Table S1. Comparison between the previous correct answers and the ones given at the Immediate post-intervention and at three months moments.

Questions	T0	T1 I	T1 C	p*	T2 I	T2 C	p*
What does the PAH acronym mean?	I	194	227	<0.001	62	97	<0.001
	C	47	267		29	94	
Which is the national Emergency number in Spain?	I	7	73	<0.001	5	17	0.35
	C	18	608		6	252	
In the Recovery Position (RP)...	I	131	218	<0.001	60	61	0.659
	C	103	242		67	92	
Which is the proper position for the first aider's hands in cardiac massage?	I	33	182	<0.001	14	47	<0.001
	C	39	440		18	201	
How would you verify whether the victim is breathing or not?	I	9	79	<0.001	4	28	0.045
	C	32	574		14	234	

If you come across an unconscious person, which of the following measures should you implement first?	**I**	133	305	<0.001	54	96	<0.001
	**C**	56	200		34	96	
Chest compressions are effective if:	**I**	165	197	<0.001	47	80	<0.001
	**C**	56	256		25	128	
The first manoeuvre for a person that is coughing and choking is:	**I**	235	306	<0.001	96	118	<0.001
	**C**	31	123		20	46	
An unconscious person that is breathing needs:	**I**	89	194	<0.001	27	65	<0.001
	**C**	75	336		25	163	
If you have initiated CPR, when should you stop?	**I**	212	314	<0.001	89	106	<0.001
	**C**	41	127		25	60	

C = Correct; I = Incorrect.
The values are expressed as absolute frequencies of paired answers (I/C). The rows correspond to the post-intervention assessment (T1) and the column to the at 3 months assessment (T2). The differences were analysed by means of the McNemar test, exclusively based on the discordant pairs (I→C y C→I).
p-values<0.05 were considered statistically significant.

Table S2. Percentage of correct answers given to each of the questionnaire questions, by teaching level (LSE-HSE).

T0	T1	T2

	LSE (%)	HSE (%)	p	LSE (%)	HSE (%)	p	LSE (%)	HSE (%)	p
What does the PAH acronym mean?	39.6	62.9	<0.001	63.4	78.3	<0.001	64.0	72.1	62
Which is the national Emergency number in Spain?	88.1	93.8	0.01	95.4	99.1	0.012	95.1	96.2	574
In the Recovery Position (RP)...	51.0	54.4	0.337	60.7	75.8	<0.001	49.4	59.2	32
Which is the proper position for the first aider's hands in cardiac massage?	67.3	79.5	0.001	85.8	96.8	<0.001	82.0	94.0	<0.001
How would you verify whether the victim is breathing or not?	86.5	92.3	0.013	92.9	97.7	0.009	90.2	96.2	0.015
If you come across an unconscious person, which of the following measures should you implement first?	37.8	42.9	0.156	71.6	71.7	0.977	63.1	72.3	0.035
Chest compressions are effective if:	46.1	60.2	<0.001	56.1	84.0	<0.001	64.3	82.1	<0.001
The first manoeuvre for a person that is coughing and choking is:	20.3	29.3	0.003	57.4	72.3	<0.001	50.6	62.0	0.013
An unconscious person that is breathing needs:	54.6	71.0	0.001	72.5	85.4	<0.001	77.7	89.7	<0.001
If you have initiated CPR, when should you stop?	25.7	30.1	0.168	55.0	79.5	<0.001	47.6	77.2	<0.001

p-values<0.05 were considered statistically significant.

Table S3. Comparison between correct answers at the Immediate post-intervention and at three months moments.

		T2		
**Questions**	**T1**	**I**	**C**	**p***
What does the PAH acronym mean?	**I**	36	30	0.085

	C	46	124	
Which is the national Emergency number in Spain?	I	0	3	0.008
	C	15	216	
In the Recovery Position (RP)...	I	61	40	0.251
	C	52	80	
Which is the proper position for the first aider's hands in cardiac massage?	I	7	21	0.766
	C	24	181	
How would you verify whether the victim is breathing or not?	I	2	10	0.424
	C	15	206	
If you come across an unconscious person, which of the following measures should you implement first?	I	32	43	0.752
	C	47	111	
Chest compressions are effective if:	I	35	44	0.015
	C	23	131	
The first manoeuvre for a person that is coughing and choking is:	I	43	27	0.003
	C	55	108	
An unconscious person that is breathing needs:	I	16	42	0.195
	C	30	145	
If you have initiated CPR, when should you stop?	I	57	22	0.002
	C	49	105	

C = Correct; I = Incorrect.

The values are expressed as absolute frequencies of paired answers (I/C). The rows correspond to the post-intervention assessment (T1) and the column to the at 3 months assessment (T2). The differences were analysed by means of the McNemar test, exclusively based on the discordant pairs (I→C y C→I).
p-values<0.05 were considered statistically significant.

Table S4. Net difference of discordant answers (T1-T2)

	T1-T2	
**Questions**	**Net change (%)**	**p***
What does the PAH acronym mean?	-6.8	0.085
Which is the national Emergency number in Spain?	-5.1	0.008
In the Recovery Position (RP)...	-5.2	0.251
Which is the proper position for the first aider's hands in cardiac massage?	-1.3	0.766

How would you verify whether the victim is breathing or not?	-2.1	0.424
If you come across an unconscious person, which of the following measures should you implement first?	-1.7	0.752
Chest compressions are effective if:	9.0	0.015
The first manoeuvre for a person that is coughing and choking is:	-12.0	0.003
An unconscious person that is breathing needs:	5.2	0.195
If you have initiated CPR, when should you stop?	-11.6	0.002

The results are presented as the net difference of discordant answers
(% of students that improved - % of students that fell back).
The comparisons were analysed by means of the McNemar test, exclusively based on the discordant pairs.
p-values<0.05 were considered statistically significant.

Table S5. PCR quality. Mean score by item and comparison between academic years.

		LSE				HSE	
		1 (n=23)	2 (n=11)	3 (n=14)	4 (n=13)	1 (n=30)	2 (n=14)
Age	Mean	11.9	13	14.6	15.4	16	17.1
Overall score	Median	81	96	95	96	98	97
	IQR	26	6	8	8	4	7
Release	Median	98	100	98	100	89.5	89.5
	IQR	8	8	40	17	17	16
Depth	Median	89	99	99	89	100	99
	IQR	29	8	4	23	1	5
Frequency	Median	119	117	119	113	114	115.5
	IQR	22	13	14	9	10	20
Rate	Median	40	49	54.5	78	81.5	85
	IQR	53	55	64	54	39	50
CCF	Median	100	100	100	100	100	100
	IQR	4	0	0	0	0	0
Categorized total score	Optimum	46	79	81	79	87	88
	Acceptable	23	7	13	21	3	13
	Needs to be improved	31	14	6	0	10	0

The measurements were made in an interval lasting approximately 60 seconds.

IQR = Interquartile Range.
Overall score: Overall quality of the PCR manoeuvres performed (the calculation considers all the subcomponents). Overall score: Percentage corresponding to the overall quality of the PCR manoeuvres performed (the calculation considers all the subcomponents).
Release: Percentage of compressions in which the chest was allowed to completely return to its normal position (Full release/Chest recoil).
Depth: Percentage of compressions that reached the proper depth (at least 5-6 cm in adults).
Frequency: Frequency in a minute.
Rate: Percentage of compressions within the target frequency range.
CCF (Chest Compression Fraction): Percentage of time devoted to performing compressions in relation to the total time.
